# Cholesterol Is a Dose-Dependent Positive Allosteric Modulator of CCR3 Ligand Affinity and G Protein Coupling

**DOI:** 10.3389/fmolb.2021.724603

**Published:** 2021-08-20

**Authors:** Evan van Aalst, Benjamin J. Wylie

**Affiliations:** Department of Chemistry and Biochemistry, Texas Tech University, Lubbock, TX, United States

**Keywords:** GPCR, CCR3, chemokine receptor, CCL11, cholesterol, G protein, lipid allostery, codon harmonization, GPCR

## Abstract

Cholesterol as an allosteric modulator of G protein-coupled receptor (GPCR) function is well documented. This quintessential mammalian lipid facilitates receptor–ligand interactions and multimerization states. Functionally, this introduces a complicated mechanism for the homeostatic modulation of GPCR signaling. Chemokine receptors are Class A GPCRs responsible for immune cell trafficking through the binding of endogenous peptide ligands. CCR3 is a CC motif chemokine receptor expressed by eosinophils and basophils. It traffics these cells by transducing the signal stimulated by the CC motif chemokine primary messengers 11, 24, and 26. These behaviors are close to the human immunoresponse. Thus, CCR3 is implicated in cancer metastasis and inflammatory conditions. However, there is a paucity of experimental evidence linking the functional states of CCR3 to the molecular mechanisms of cholesterol–receptor cooperativity. In this vein, we present a means to combine codon harmonization and a maltose-binding protein fusion tag to produce CCR3 from *E. coli*. This technique yields ∼2.6 mg of functional GPCR per liter of minimal media. We leveraged this protein production capability to investigate the effects of cholesterol on CCR3 function *in vitro*. We found that affinity for the endogenous ligand CCL11 increases in a dose-dependent manner with cholesterol concentration in both styrene:maleic acid lipid particles (SMALPs) and proteoliposomes. This heightened receptor activation directly translates to increased signal transduction as measured by the GTPase activity of the bound G-protein *α* inhibitory subunit 3 (G*α*_i_3). This work represents a critical step forward in understanding the role of cholesterol-GPCR allostery in regulation of signal transduction.

## Introduction

**FIGURE 1:**
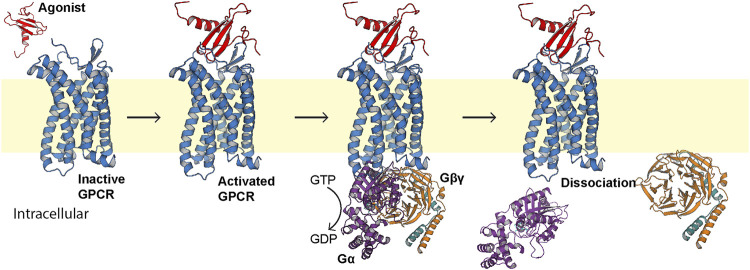
Generalized first step of GPCR signal transduction. The extracellular agonist (red, PDB ID 1EOT) (Crump et al., 1998) binds to the orthosteric pocket of the GPCR (blue) to elicit a conformational change recognized intracellularly by the heterotrimeric G protein (PDB ID 1GP2) (Wall et al., 1995). The G protein binds, exchanges bound GDP for GTP, and the *α* subunit (purple) dissociates from the βγ subunits (orange and teal, respectively).

G protein-coupled receptors (GPCRs) are integral membrane proteins comprising a canonical seven-transmembrane alpha helical architecture (Rosenbaum et al., 2009). In response to external stimuli, this helical bundle undergoes a conformational change that is recognized by an intracellular heterotrimeric G protein (Kim et al., 2013). This molecular recognition event leads to an exchange of bound GDP for GTP in the G protein, triggering dissociation of the *α* and *βγ* subunits (Figure 1) (Sullivan et al., 1987). The *α* subunit then acts as an effector to influence downstream events such as modulation of adenylate cyclase functionality (Federman et al., 1992), while the *βγ* subunit can trigger cleavage of phosphatidylinositol-(4,5)-bisphosphate (PIP_2_) (Katz et al., 1992) and ion channel activation (Pegan et al., 2005; Nishida et al., 2007).

Functional interplay between lipid constituents and membrane proteins is well documented. We previously reported that the bacterial K^+^ channel KirBac1.1 orders the membrane (Borcik et al., 2019) and that the membrane activates structural transitions and associated biological functions (Amani et al., 2020; Borcik et al., 2020). Like K^+^ channels, GPCRs are regulated by lipids through both direct allosteric interactions and changes to membrane mechanical and thermodynamic properties (Botelho et al., 2006). Perhaps the most widely studied of these functional lipids is cholesterol (Jafurulla et al., 2019), although allosteric effects of phosphoserines (Dawaliby et al., 2016), sphingolipids (Chattopadhyay, 2014), phosphoinositols like PIP_2_ (Yen et al., 2018), and the binding synergy between multiple lipid species (Xu et al., 2021) are of increasing interest. A canonical Class A GPCR cholesterol consensus motif (CCM) of (R,K)^4.39–4.43^–(W,Y)^4.50^–(I,V,L)^4.46^–(F,Y)^2.41^ has been identified in the *β*_2_ adrenergic receptor (*β*_2_AR), following the Weinstein–Ballesteros numbering convention (Ballesteros and Weinstein, 1995; Hanson et al., 2008). However, only 21% of Class A receptors contain this sequence (Hanson et al*.*, 2008), and this motif is conspicuously absent in chemokine receptors (Legler et al., 2017). Furthermore, it is observed that such cholesterol binding motifs are not necessarily occupied even when present (Marlow et al., 2021). Cholesterol has, nevertheless, still been implicated as an allosteric modulator of chemokine receptor function (Zhukovsky et al., 2013; Pluhackova et al., 2016; Legler et al., 2017; Gahbauer et al., 2018; Calmet et al., 2020).

Chemokine receptors are a subclass of Class A GPCRs. They trigger cellular trafficking of immune cells in response to chemotactic cytokine (chemokine) ligands (Stone et al., 2017). Perhaps the most well-known chemokine receptors are CC motif chemokine receptor 5 (CCR5) and CXC motif chemokine receptor 4 (CXCR4), which act as coreceptors for HIV infection (Deng et al., 1996; Ma et al., 1998). Although the CCM identified in *β*_2_AR is absent from chemokine receptors, experimental evidence indicates receptor–lipid interactions, often driving receptor dimerization (Pluhackova et al., 2016; Legler et al., 2017; Gahbauer et al., 2018; Calmet et al., 2020). However, there are many outstanding questions regarding the direct and indirect influence of cholesterol on function *in vivo*. Much of what is known is derived from crystal structure co-crystallization and molecular dynamics (MD) simulations. While these studies are foundational, the conclusions are not definitive and experimental functional data are needed.

**FIGURE 2:**
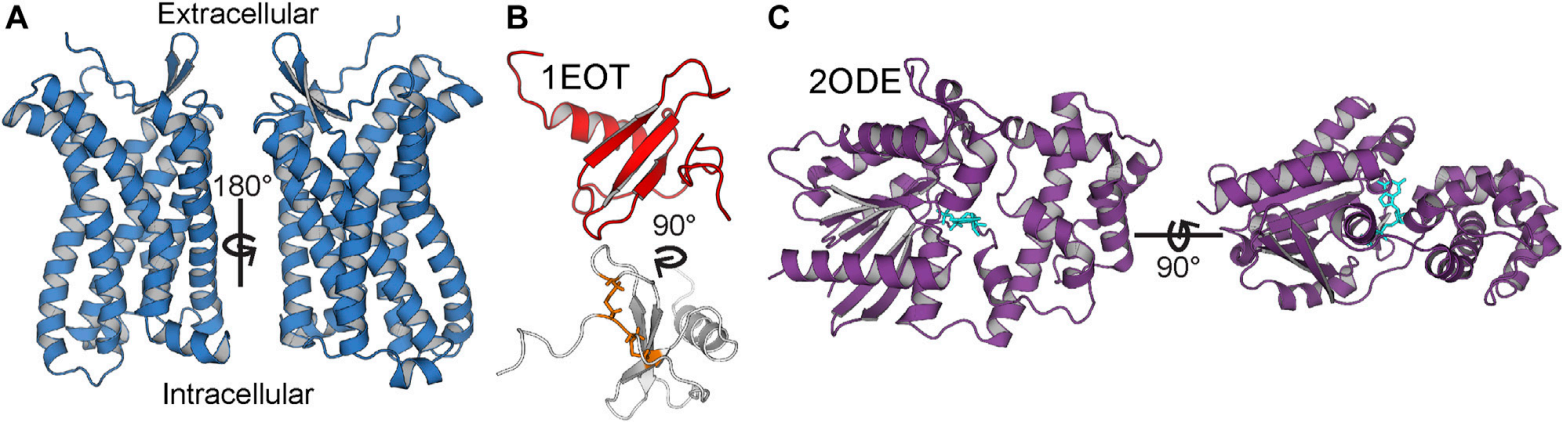
Anatomy of CCR3, CCL11, and Gα_i_3. **(A)** CCR3 homology model is marked by the canonical seven-transmembrane helical architecture. **(B)** Solution state NMR structure of CCL11 (PDB ID 1EOT) (Crump et al., 1998) shows the structural disulfide bonds in orange (bottom). **(C)** Gα_i_3 (purple) with bound GDP (cyan) from the crystal structure of Gα_i_3 bound to the regulator of G protein signaling 8, RGS8 (PDB ID 2ODE) (Soundararajan et al., 2008).

CC motif chemokine receptor 3 (CCR3, Figure 2A) is a Class A GPCR mainly expressed by eosinophils. Like all chemokine receptors, its primary messengers are endogenous peptide ligands. Specifically, CCL11, CCL24, and CCL26 (eotaxins 1, 2, and 3, respectively; CCL11 is depicted in Figure 2B) activate CCR3 and trigger chemotaxis of the expressing cell (Ge et al., 2015). This occurs through the G protein inhibitory *α* subunit (Gα_i_, Figure 2C), which triggers downstream inhibition of adenylate cyclase (Kitaura et al., 1996). It is, however, unclear as to which of the 3 G*α*_i_ subunits is primarily involved in the signaling cascade or if there is significant promiscuity *in vivo* between CCR3 and Gα_i_s 1, 2, and 3. Moreover, the influence of direct lipid allostery on CCR3-G protein coupling and signal transduction is undocumented.

Given its role in leukocyte trafficking, CCR3 provokes inflammatory conditions such as asthma (Gauvreau et al., 2018), rheumatoid arthritis (Katschke et al., 2001), and eosinophilic esophagitis (Dunn et al., 2020). Furthermore, CCR3 is correlated with heightened invasive potential of metastatic liver (Jin et al., 2017), prostate (Ishida et al., 2018), and kidney cancers (Johrer et al., 2005). It is also a coreceptor for some strains of HIV (He et al., 1997). As a result, CCR3 is an attractive therapeutic target. However, comparatively little is known about the structural biology of CCR3 with respect to other chemokine receptors such as CCR5 and CXCR4. CCR3’s natural agonists help regulate the relative monomer–dimer higher-order oligomer populations *in vivo* (Song et al., 2018), but the influence of cholesterol on this interaction is unknown. Therefore, investigation of the structure–function relationship and the lipid agency is an attractive and necessary long-term goal.

In pursuit of this goal, we implemented a codon harmonization scheme we previously reported to positively influence heterologous membrane protein yield (van Aalst et al., 2020). This technique is called DNA codon usage for measured base optimization, or DUMB optimization (DO). Codon harmonization aims to site-specifically modify the codons comprising the heterologous construct to match the codon usage frequency of the native organism more closely in order to improve cotranslational folding (Angov et al., 2008; Rodriguez et al., 2018). This increases the fraction of protein that is properly folded, membrane-inserted, and functional by slowing translation through the introduction of targeted rare codons. Through implementation of DUMB optimization and incorporation of a maltose-binding protein (MBP) solubility tag (Ge et al., 2015) in our construct, we report the heterologous production of CCR3 after tag removal at yields of ∼2.5–2.8 mg/L from M9 minimal media (Bhate et al., 2013). CCL11 and G*α*_i_3 were DUMB optimized as a matter of course in expression optimization, attaining ∼2.6 ± 0.3 and 15.1 ± 0.3 mg/L, respectively, for each, from M9 minimal media. We describe the positive cooperativity between membrane cholesterol and CCR3 binding affinity to its endogenous ligand CCL11, quantified *via* a fluorescence polarization assay (Rossi and Taylor, 2011) in lipid environments of increasing cholesterol content. We then confirm that this cholesterol-induced modulation of ligand affinity translates to increased signal transduction, measured *via* coupling to and activation of G*α*_i_3 GTPase. This is, to our knowledge, the first experimental evidence of cholesterol-receptor interactions and their effect on ligand affinity and the efficacy of signal transduction catalyzed by CCR3.

## Materials and Methods

### Construct Design and Gene Insertion

The human CCR3 amino acid sequence was obtained from the UniProt database (P51677). The human CCL11 amino acid sequence was obtained from the UniProt database (P51671) and truncated to residues 24–97 to remove the propeptide. The human G *α* inhibitory 3 (G*α*_i_3) amino acid sequence was obtained from the UniProt database (P08754). The amino acid sequences were reverse engineered into fully optimized (FO) DNA sequences according to *E. coli* codon usage, serving as a platform for the application of DUMB optimization. Here, the FO constructs use codons to transcribe the proteins that correspond to only the most abundantly found tRNAs in the expression vector.

**FIGURE 3:**
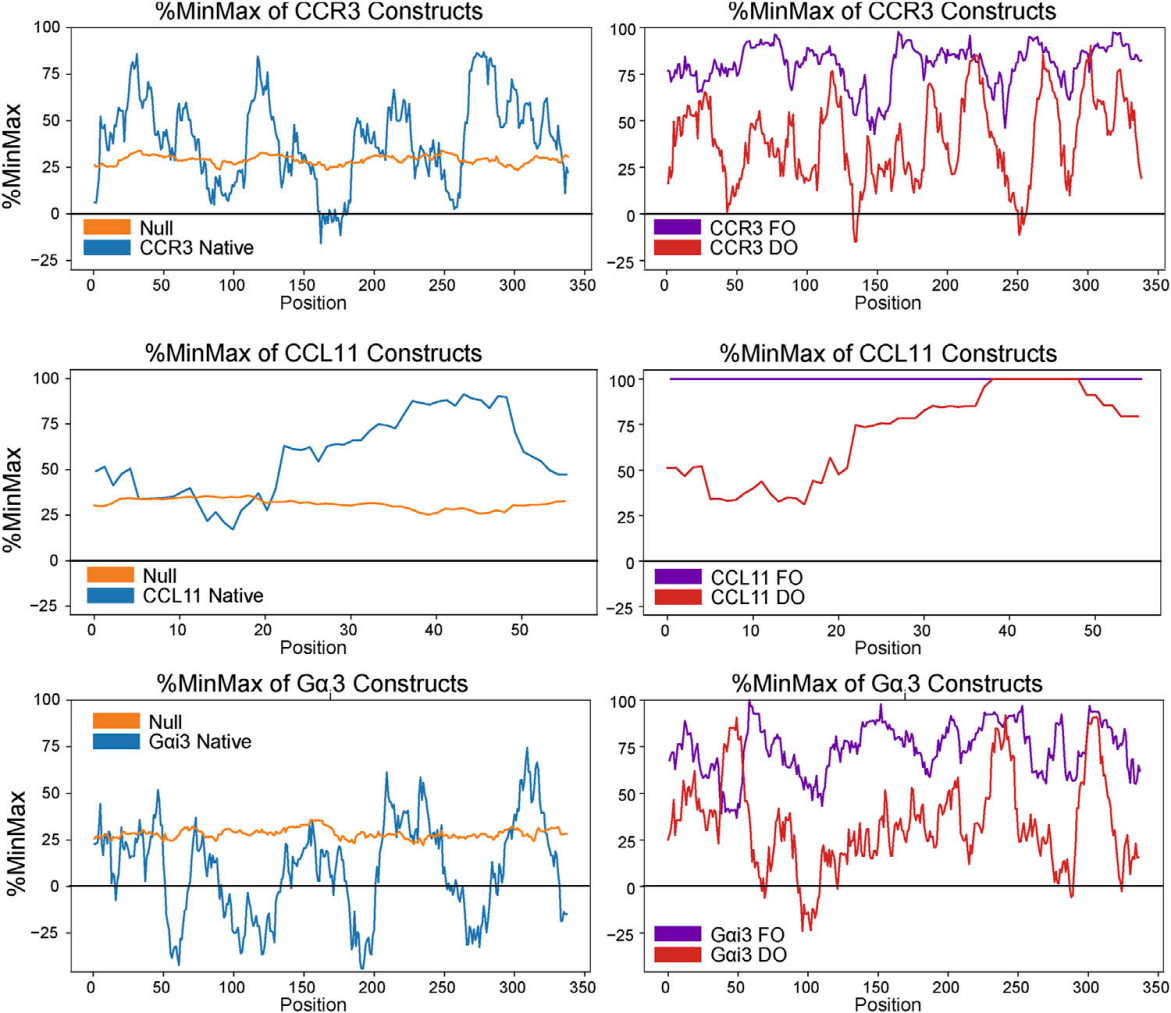
%MinMax (Rodriguez et al., 2018) of native sequences compared to optimized heterologous sequences (FO) and constructs codon-harmonized through DUMB optimization (DO). In general, %MinMax of the DO sequences (red) more closely resemble the native values (blue) than the FO values (purple).

The native human CCR3 DNA sequence was obtained from the European Nucleotide Archive (ENA, Sequence: U51241) and codon-harmonized according to DUMB optimization (DO) for expression in *E. coli*, as previously described (van Aalst et al*.*, 2020). Briefly, codons were replaced in the expression sequence to match the codon usage frequencies found within the native sequence as *E. coli* codon usage permitted. Substitution was performed such that no alternative codons in the host system were chosen with a relative usage below 5% of the native usage frequency. Human and *E. coli* codon usage frequencies from the Graphical Codon Usage Analyzer (http://gcua.schoedl.de/) were used in designing the DO gene sequence (Fuhrmann et al., 2004). For more information on the codon harmonization process, see reference van Aalst et al., 2020. %MinMax analysis (Rodriguez et al*.*, 2018) of the native, FO, and DO sequences visualizes the extent of deoptimization (Figure 3). The native human G*α*_i_3 (Sequence: J03005.1) and human CCL11 (D49372.1) gene sequences were also obtained from the ENA and codon-harmonized in the same way as a matter of course in the optimization process.

CCR3, CCL11, and G*α*_i_3 gene sequences were ordered from GeneArt (Thermo Fisher). The expression vector pMAL-p4x was ordered from Addgene. 5′ EcoRI and 3’ Hind III restriction enzymes (Thermo Scientific) were used to clone each sequence into the vector downstream of MalE. This resulted in an N–maltose-binding protein (MBP)-8x His tag–TEV site–CCL11–C construct, in the case of CCL11. For CCR3, the initial FO construct that was described (Ge et al., 2015) was DUMB optimized, and a GGGGS 4x repeat between the TEV site and the *N* terminus of CCR3 was added to promote cleavage (Chen et al., 2013). This resulted in an N–maltose-binding protein (MBP)-8x His tag–TEV site–(GGGGS)_4_–CCR3–C construct. The restriction enzymes NcoI and BamHI (Thermo Scientific) were used to insert the G*α*_i_3 sequence into the expression vector pET-28a (+) (Novus Biologicals). This resulted in an N–Met-Gly–8x His–TEV site–Gα_i_3–C construct. In all cases, gene insertion and sequence conservation were verified by sequencing.

### CCR3 Expression and Purification

*E. coli* C43 (DE3) cells harboring the pMAL-p4x-CCR3 plasmid were grown in M9 minimal media containing 2 mM MgSO4, 0.1 mM CaCl2, 100 μg/ml ampicillin, 3 ml of 100x minimum essential vitamin stock, 96.22 mM Na_2_HPO_4_, 44.1 mM K_2_HPO4, 17.1 mM NaCl, 5 g glucose per L (0.5% w/v), 3.75 g NH_4_Cl per L (0.375% w/v), and 20 ml of Solution C (Supplementary Table S1) per L. Cultures were grown at 220 rpm and 37°C until an OD_600_ of ∼1.0 was reached. The cultures were cooled to 18°C and induced with 1 mM IPTG for 24 h. Cell cultures were then pelleted at 5,500 rpm for 10 min and stored at −80°C for future use.

Cell pellets were removed from storage at −80°C and resuspended in 5 ml of lysis cocktail per g of cells (20 mM HEPES, pH 8.0, 150 mM KCl, 0.02% NaN_3_, 10 mM MgSO_4_, 0.2 mg/ml lysozyme, 0.2 mg/ml RNase A, 1 mM phenylmethylsulfonyl fluoride (PMSF), and 1 mM benzamidine). PIERCE EDTA-free protease inhibitor tablets (Thermo Fisher) were added at one tablet per 6 g of cells. Cells were lysed *via* homogenization, and protein was extracted from membranes *via* addition of n-Dodecyl-β-D-Maltoside (DDM, Anatrace) and cholesteryl hemisuccinate Tris-salt (CHS, Anatrace) to final concentrations of 20 and 2 mM, respectively. Extraction took place overnight at 4°C with rocking. The solution was centrifuged at 125,000 g for 40 min at 4°C to remove cell debris. The supernatant was filtered through a 0.22-μm PES bottle top filter and loaded onto a 5-ml nickel affinity column (NAC, GE Healthcare Life Sciences) equilibrated in wash buffer (20 mM HEPES, pH 8.0, 150 mM KCl, 10 mM imidazole, 0.02% NaN_3_, 2.5 mM DDM, and 0.25 mM CHS). The column was then treated with five column volumes of wash buffer before elution with five column volumes of elution buffer (20 mM HEPES, pH 8.0, 150 mM KCl, 250 mM imidazole, 0.02% NaN_3_, 2.5 mM DDM, and 0.25 mM CHS).

Eluted protein was transferred into cleavage buffer (20 mM HEPES, pH 8.0, 150 mM KCl, 1 mM EDTA, 1 mM DDM, 0.1 mM CHS, and 0.5 mM DTT) using a desalting column (GE Healthcare Life Sciences) equilibrated with cleavage buffer. Tobacco etch virus (TEV) protease (Kapust et al., 2001) was added from glycerol stocks at a typical ratio of 1 mg TEV to 3 mg MBP-CCR3 with 1 mM DTT and set to rock overnight at 4°C. Samples were then transferred into wash buffer to remove EDTA and subjected to the NAC. Flow-through containing cleaved CCR3 was collected and the elution peak containing TEV, the MBP tag, and uncleaved MBP-CCR3 was discarded. The flow-through was then concentrated to ∼2 mg/ml using an Amicon Stirred Cell with Ultracel 30 kDa Ultrafiltration Discs (Millipore) before loading onto a HiLoad 16/600 Superdex Prep grade 200 column (GE Healthcare Life Sciences) equilibrated in exchange buffer. Cleaved CCR3 fractions were pooled, concentration was determined using optical density at 280 nm, and samples were stored at 4°C for future use.

### CCL11 Expression and Purification

pMAL-p4x harboring CCL11 was cultured in M9 minimal media at 220 rpm and 37°C until an OD_600_ of ∼0.8 was reached. The cultures were cooled to 18°C and induced with 0.5 mM IPTG for 24 h. Cell aliquots were centrifuged at 5.5 k rpm for 10 min, and the resulting pellets were stored at −80°C for future use.

Cell pellets were resuspended in CCL11 wash buffer (20 mM HEPES, pH 7.5, 300 mM KCl, 10 mM Imidazole, and 0.02% NaN_3_) supplemented with 0.2 mg/ml lysozyme, 0.2 mg/ml RNase A, 1 mM PMSF, 1 mM benzamidine, and 1 PIERCE EDTA-free protease inhibitor tablet per 6 g of cells. Cells were lysed *via* triplicate passage through a homogenizer. Cell debris was centrifuged at 17,000 rpm for 30 min followed by clarification of the lysate using a 0.22-μm PES bottle top filter. The clarified lysate was loaded onto an NAC preequilibrated in CCL11 wash buffer, washed five times with the same, and eluted with five column volumes of CCL11 elution buffer (20 mM HEPES, pH 7.5, 300 mM KCl, 250 mM imidazole, and 0.02% NaN_3_). The elution peak was then transferred back into CCL11 wash buffer using a desalting column for the reverse NAC.

The TEV was added in a typical ratio of 1 mg TEV per 4 mg MBP-CCL11 and set to cleave at 4 °C with rocking for 1 h. The cleavage mixture was then passed through the NAC, and the flow-through containing cleaved CCL11 was collected. This was concentrated to ∼5 ml using an Amicon Stirred Cell with Ultracel 3 kDa Ultrafiltration Discs (Millipore) and loaded onto a HiLoad 16/600 Superdex Prep grade 75 column equilibrated in CCL11 exchange buffer (20 mM HEPES, pH 7.5, 300 mM KCl, 1 mM EDTA, and 0.02% NaN_3_). The CCL11 elution fractions were collected, and concentration was determined *via* optical density at 280 nm.

### G*α*_i_3 Expression and Purification

*E. coli* BL21 codon + (DE3) cells harboring the pET-28a (+) G*α*_i_3 plasmid were grown in M9 minimal media at 220 rpm and 37°C until an OD_600_ of ∼1.0 was reached. The cultures were cooled to 20°C and induced with 0.5 mM IPTG for 24 h. Cell cultures were then pelleted at 5,500 rpm for 10 min and stored at −80°C for future use.

Cell pellets were resuspended in G*α*_i_3 wash buffer (20 mM HEPES, pH 7.5, 150 mM KCl, 10 mM Imidazole, 10 μM GDP, 2.5 mM MgCl_2_, and 0.02% NaN_3_) supplemented with 0.2 mg/ml lysozyme, 0.2 mg/ml RNase A, 1 mM PMSF, 1 mM benzamidine, and 1 PIERCE EDTA-free protease inhibitor tablet per 6 g of cells. Cells were lysed *via* triplicate passage through a homogenizer. Cell debris was centrifuged at 17,000 rpm for 30 min, followed by clarification of the lysate using a 0.22-μm PES bottle top filter. The clarified lysate was loaded onto an NAC preequilibrated in wash buffer, washed five times with the same, and eluted with five column volumes of elution buffer (20 mM HEPES, pH 7.5, 150 mM KCl, 250 mM Imidazole, 10 μM GDP, 2.5 mM MgCl_2_, and 0.02% NaN_3_). The elution peak was then loaded onto a HiLoad 16/600 Superdex Prep grade 75 column equilibrated in G*α*_i_3 exchange buffer (20 mM HEPES, pH 7.5, 150 mM KCl, 10 μM GDP, 2.5 mM MgCl_2_, and 0.02% NaN_3_). The elution fractions were collected, and concentration was determined *via* optical density at 280 nm.

### SDS-PAGE Analysis

Samples were combined at a ratio of 1:1 with 2x Laemmli buffer (20% glycerol, 125 mM Tris HCl pH 6.8, 4% sodium dodecyl sulfate (SDS), 0.02% bromophenol blue) for denaturation (Laemmli, 1970). Samples were then loaded into a Mini-PROTEAN TGX precast any-kD 10-well gel (Bio-Rad) with a Precision Plus Dual Standard protein ladder (Bio-Rad). The gel was run in running buffer (192 mM glycine, 25 mM, 0.1% SDS) for 53 min to remove loading dye at 400 mA and 150 V on a PowerPac Basic module (Bio-Rad). The gel was removed from the casing and stained in staining buffer (20% methanol and 10% acetic acid with 1 mg/ml Coomassie R250) with orbital rotation at 69 rpm until the gel was no longer visible. The gel was then destained in destaining buffer (20% methanol and 10% acetic acid).

### Circular Dichroism Analysis

CCR3 was buffer-exchanged into CCR3 CD buffer (5 mM Tris-HCl, pH 8.0, 50 mM sodium acetate, 1 mM DDM, and 0.1 mM CHS) and concentrated to 0.3 mg/ml (7.2 μM). Immediately prior to analysis, buffer and CCR3-containing samples were diluted 4x to allow for data acquisition. Spectra were acquired using a J-815 CD spectrophotometer (JASCO Co., Easton, MD, United States) at 22°C in the spectral range of 180–260 nm at a rate of 1 nm/sec and a path length of 0.1 cm. 10 spectra each of the diluted buffer blank and CCR3 samples were recorded and averaged. Background spectra of the buffer were acquired identically and subtracted from the experimental data. Spectral fitting and secondary structure analysis for CCR3 were carried out using the DichroWeb (Whitmore and Wallace, 2004; Whitmore and Wallace, 2008) server using the K2D algorithm (Andrade et al., 1993). Presented secondary structure percentages from experimental data were calculated using DichroWeb analysis and compared to a model of CCR3. This model was generated by submitting the full-length CCR3 sequence to the Baker laboratory ROBETTA comparative modeling server (Song et al., 2013), using the CCR5 crystal structure PDB 4MBS (Tan et al., 2013a) as the template because of the high sequence similarity. The model was truncated to residues 23–317, and all truncated residues and the GGGGS4x linker were assumed to be random coils for percent secondary structure calculations. In addition, the CCL11 structure 1EOT (Crump et al., 1998) was docked to the CCR3 model using HADDOCK (van Zundert et al., 2016) to visualize the CCL11-bound CCR3 model observed throughout this work. Residue–residue interaction restraints upon drive docking were derived from information available at the GPCR Database (Duchesnes et al., 2006; Millard et al., 2014; Pandy-Szekeres et al., 2018). CCL11 was buffer-exchanged into 10 mM sodium phosphate buffer, pH 7.5. Data were acquired at a concentration of 18.14 μM and processed in the same manner as CCR3. Bestsel was used to fit CCL11 spectral data and predict the percent secondary structure from the experimental data to compare to the published structure 1EOT (Crump et al*.*, 1998; Micsonai et al., 2015; Micsonai et al., 2018; Micsonai et al., 2021). G*α*_i_3 was buffer-exchanged into 10 mM sodium phosphate buffer, pH 7.5, and 10 μM GDP. Spectra were acquired in the same way as before at a protein concentration of 1.85 μM. Data were fit using DichroWeb with the Contin-LL algorithm (Provencher and Glöckner, 1981) and reference set 4 (Sreerama and Woody, 2000). The extrapolated secondary structure from the experimental fit was compared to the crystal structure of activated G*α*_i_3 in complex with RGS10 (PDB ID 2IHB) (Soundararajan et al., 2008). All non-crystallizing residues were assumed to be random coils for percent secondary structure calculations.

### CCR3 Reconstitution and Formation of Polymer Discs

1-pamlitoyl-2-oleoyl-phosphatidylcholine (POPC or PC, Avanti Polar Lipids) and cholesterol (Sigma) were solvated in chloroform at 10 mg/ml, and then PC-only and 10, 20, 30, 40, and 60% cholesterol (mol%) mixtures were formed, blown down under a N_2_ stream, and dried overnight *in vacuo* to produce a lipid film. Dried films were evenly divided (one aliquot for a protein-free control) and then solvated in non-detergent buffer (NDB, 20 mM HEPES pH 8.0, 150 mM KCl, 0.02% NaN_3_, and 1 mM EDTA) supplemented with 25 mM 3-[(3-Cholamidopropyl) dimethylammonio]-1-propanesulfonate (CHAPS, Anatrace) using mild sonication at 5 mg lipid/ml. Solvated films were then set on the benchtop for 3–5 h before the addition of protein, added at a ratio of 1 mg of protein per 4 mg of lipids. An equal volume of exchange buffer was added to protein-free (PF) samples. For samples reconstituted in the presence of CCL11, the agonist was added at a molar ratio of 5:1 CCL11:CCR3, or an equal volume of CCL11 exchange buffer was added to control samples. All samples were set to anneal for 3 h on the benchtop, during which Bio-Bead SM-2 Resin (Bio-Rad) was prepared by 3x degassing washes with methanol, followed by 3x washes with DI water and resuspension in NDB. A double portion of Bio-Beads (∼60 mg) was added to each sample before nutation at room temperature. Samples were nutated in this way for 48–72 h, with ∼30 mg Bio-Beads being added twice daily until the detergent was completely removed, evidenced by increased turbidity and loss of detergent bubbles upon manual agitation. Bio-Beads were removed by centrifugation at 500 rpm using PIERCE columns to collect fully formed proteoliposomes. To form lipid particles (SMALPs) from proteoliposomal samples, 3:1 pre-hydrolyzed styrene:maleic acid (SMA) was added at 3 mg SMA per 1 mg of lipids dropwise with inversion to each sample to facilitate polymer disc formation (Lipodisq, Thermo Fisher). Proteoliposomal samples typically turned clear within moments of SMA addition. All samples were nutated overnight to ensure SMALP formation.

### Fluorescence Polarization Assays

CCL11 in CCL11 exchange buffer was concentrated to >2 mg/ml and incubated with a 4x molar excess of fluorescin isothiocyanate (FITC, Invitrogen) for 1 h at room temperature. This was achieved in the dark, using an orbital shaker at 150 rpm and a pH value of 7.5 to facilitate labeling of the *N* terminus with the fluorophore. Following this, the sample was diluted to 1–2 ml with the same buffer and exchanged back into CCL11 exchange buffer sans excess FITC using a HiPrep 26/10 desalting column. A Lowry assay (DC protein assay, Bio-Rad) was performed to gauge protein concentrations. Fluorescence polarization assays were performed at room temperature using a Biotek Synergy NEO2 fluorescent plate reader equipped with a fluorescein filter (Biotek Instruments, Winooski, VT, United States). Fluorescence polarization was calculated automatically using the instrument as follows (Rossi and Taylor, 2011):

$$P=\;\frac{I||-I⊥}{I||+I⊥}$$

where *I*|| is the observed parallel intensity, *I*⊥ is the observed perpendicular intensity, and *P* is polarization. Static concentrations of 100 nM FITC-CCL11 and 0.1 µg/µl bovine serum albumin (BSA, Thermo Scientific) for nonspecific binding were added to each assay well. CCR3 was added to the desired concentration. Protein-free SMALPs or proteoliposomes were then added to balance out the lipid/SMA material such that the concentrations were equivalent across all wells. NDB was then added to fill to 30 µl. The concentration of FITC-CCL11, BSA, and lipids/SMA were static across all conditions and replicates. Data were normalized by subtracting the lowest zero-point (no CCR3) value in a curve from each read to bring all curves within the same reference frame.

### GTP Hydrolysis Assays

G*α*_i_3 hydrolyzes GTP when bound to and activated by CCR3. Unhydrolyzed GTP is enzymatically converted to ATP and then to luminescence *via* luciferase. GTP turnover was thus quantified using a modified protocol of the GTPase-Glo™ assay (Promega) (Mondal et al., 2015) at room temperature for all steps, with a reaction incubation time of 2 h in all cases. After incubation, reconstituted GTPase-Glo™ reagent was added and incubated for 30 min at room temperature. Detection reagent was added, followed by an additional 5–10 min of incubation. Luminescence was read using a Cytation 3 multimode reader (Biotek Instruments, Winooski, VT, United States). Intrinsic GTPase activity of G*α*_i_3 was analyzed using 2.5 μM apo-G*α*_i_3 in 20 mM HEPES (pH 7.5), 150 mM KCl, 5 mM MgCl_2_, 20 mM EDTA, 0.1 mM TCEP, 10 μM GDP, and 1 or 4 μM GTP. Preliminary CCL11-induced CCR3 activation of G*α*_i_3 was analyzed in assay buffer (20 mM HEPES, pH 7.5, 150 mM KCl, 1 mM EDTA, 0.1 mM TCEP, 10 μM GDP, and 4 μM GTP) containing 5 mM MgCl_2_, 1 mM DDM, and 0.1 mM CHS. Agonist-driven GTPase activity was analyzed in SMALPs formed from POPC with 0–30% cholesterol in assay buffer with no additives. CCR3 samples reconstituted in the presence of CCL11 were analyzed in assay buffer plus 5 mM MgCl_2_. Assays in detergent, SMALPs, and proteoliposomes were performed with 5 μM CCL11, 1 μM CCR3, and 1 μM G*α*_i_3. Relative light units (RLUs) of all assay runs were blank corrected by subtracting the average of three blank replicates (buffer with no GTP and background luminescence) from each replicate. % Hydrolysis was calculated from raw data as follows:

$$\%\;\text{GTP}\;\text{Hydrolysis}=\frac{{\text{RLU}}_{std}-{\text{ RLU}}_{replicate}}{{\text{RLU}}_{std}}\times 100\;$$

## Results

### Codon Harmonization and Maltose-Binding Protein Facilitate Heterologous Protein Production

The total yield of functional, folded protein is the main bottleneck in the study of GPCRs. Here, we introduced a maltose-binding protein (MBP) fusion tag to aid in protein folding and solubility, as previously described (Ge et al., 2015). Furthermore, we employed codon harmonization, a method to optimize heterologous plasmid DNA sequences (Angov et al., 2008). We previously showed our codon harmonization strategy, dubbed DNA codon usage for measured base optimization, or DUMB optimization (DO), which dramatically increased both the yield and activity of an exogenously expressed chimeric membrane protein (van Aalst et al., 2020). Results of %MinMax analysis of proteins in this work show the codon usage of the fully optimized and codon-harmonized constructs as compared to native usage (Figure 3). Codon usage is presented as a sliding window of 21 codons to visualize the extent of optimization or deoptimization of each gene sequence to compare native usage (blue) to a random reverse transcription control (orange), FO constructs (purple), and DO constructs (red). These techniques facilitate the production of 2.6 ± 0.2 mg/L (*n* = 3, ± σ) of full length and functional WT CCR3 (Figure 2A), after proteolytic cleavage of the MBP tag, from M9 minimal media. It is expected that an even greater yield would be observed if the expression cultures were grown in rich media. CCL11 and G*α*_i_3 were DUMB optimized as a matter of course in workflow optimization and produced yields of ∼2.6 ± 0.3 and 15.1 ± 0.3 mg/L, respectively, from minimal media.

### Characterization of CCR3, CCL11, and G*α*_i_3 Secondary Structure

**FIGURE 4:**
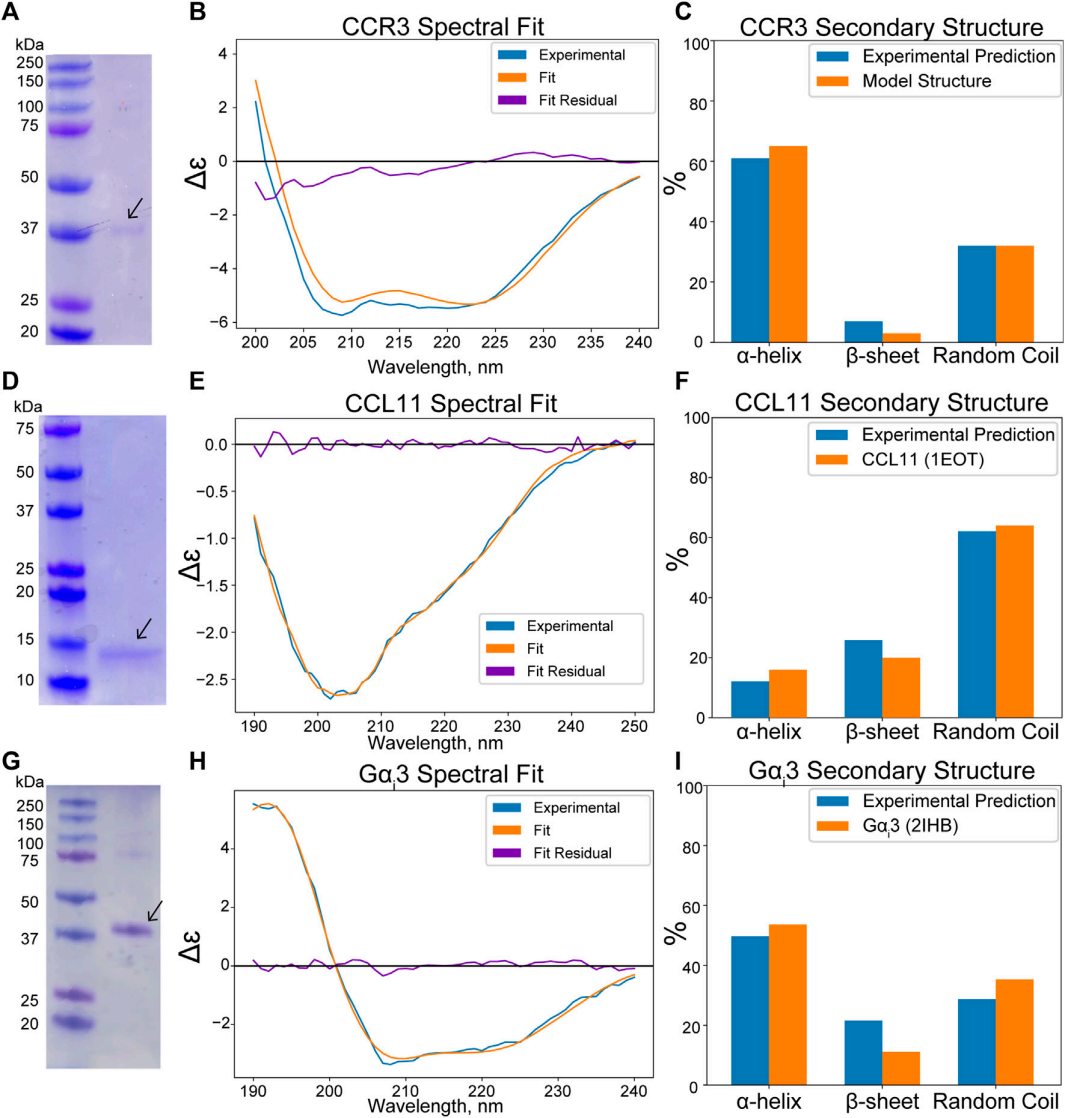
(Wavelength, nm x axis label in panel H) was cut off. **(A)** SDS-PAGE analysis of CCR3 reveals a ∼37-kDa band, consistent with the predicted molecular weight of 42.4 kDa. **(B)** Experimental CD spectra of CCR3 (blue) compared to the DichroWeb (Whitmore and Wallace, 2004) experimental fit (orange) and the residual fit (purple). **(C)** Quantitative assessment compared to the CCR3 model suggests a high degree of helical content. **(D)** SDS-PAGE analysis of CCL11 reveals an apparent molecular weight of ∼14 kDa, consistent with previous observations. **(E)** CD spectra of CCL11 (blue) compared to the fit (orange) and the residual fit (purple). The spectrum was fit using Bestsel (Micsonai et al., 2015; Micsonai et al., 2018; Micsonai et al., 2021). **(F)** Bestsel-predicted percent secondary structure for the CCL11 construct compared to the NMR structure (PDB ID 1EOT) (Crump et al., 1998). **(G)** SDS-PAGE analysis of Gα_i_3 shows good agreement with the predicted construct molecular weight of 42.7 kDa. **(H)** CD spectra of Gα_i_3 (blue) compared to the DichroWeb experimental fit (orange) and the residual fit (purple). **(I)** Comparison of the experimentally predicted % secondary structure to that found in the crystal structure (PDB ID 2IHB) (Soundararajan et al., 2008).

An apparent band slightly above the 37-kDa marker is visible in the SDS-PAGE analysis of CCR3, consistent with the predicted molecular weight of 42.4 kDa with the linker sequence in our construct (Figure 4A) after MBP cleavage and size exclusion chromatography (SEC) elution (Supplementary Figure S1A). This band conforms to previously reported SDS-PAGE analysis of similar CCR3 constructs in which CCR3 was observed to form SDS-resistant dimers (Wang et al., 2013; Ge et al., 2015). This could explain the faint bands observed at roughly 80, 120, and 160 kDa. Full gel images are available in the Supplementary Material (Supplementary Figure S2). Circular dichroism (CD) analysis reveals deep wells at 208 and 220 nm, consistent with a highly *α*-helical protein (Figure 4B). Experimental CD data were fit using DichroWeb (Whitmore and Wallace, 2004; Whitmore and Wallace, 2008). The K2D algorithm (Andrade et al., 1993) was selected over the Contin-LL algorithm (Provencher and Glöckner, 1981) paired with the SMP180 reference set (Abdul-Gader et al., 2011) due to differences in the normalized RMSD (NRMSD, 0.122 vs. 0.365, respectively). NRMSD values between 0.1 and 0.2 generally suggest similarity between experimental and fit secondary structures, whereas values greater than 0.2 indicate little resemblance (Mao et al., 1982). Predicted secondary structures derived from DichroWeb also show good agreement between experimental and model data when model truncated residues are assumed to be random coils (Figure 4C). Interestingly, the small observed *β*-sheet amount in the model (Figure 2A), derived from the CCR5 crystal structure template (Tan et al., 2013b), is conserved in the experimental CD spectrum according to the fitting.

CCL11 (Figure 2B) is also produced as an MBP fusion construct in *E. coli* to facilitate formation of the structural disulfide bonds in the more oxidizing periplasmic space. While this has a detrimental effect on yield, it consistently produces properly folded protein. An apparent band of ∼14 kDa is observed for our CCL11 construct after MBP tag removal and subsequent SEC elution (Figure 4D; Supplementary Figure S1B). This is larger than the expected 8.4 kDa but is consistent with previously reported CCL11 constructs (Mingqing Wang, 2014). CD analysis of our construct confirms the conservation of the typical chemokine fold (Figure 4E, NRMSD 0.01858). The secondary structure distributions predicted from the fit using Bestsel (Micsonai et al., 2015; Micsonai et al., 2018; Micsonai et al., 2021) are consistent with the solution state NMR structure 1EOT (Crump et al., 1998) (Figure 4F).

The G*α*_i_3 subunit (Figure 2C) is expressed as an *N* terminal His-tagged construct. The theoretical molecular weight of our construct after SEC elution (Supplementary Figure S1C) is ∼42.7 kDa, consistent with the apparent SDS-PAGE band (Figure 4G). The CD spectrum was fit using DichroWeb using the Contin-LL algorithm (Provencher and Glöckner, 1981) and spectral reference set 4 (Sreerama and Woody, 2000) (Figure 4H, NRMSD 0.045). Comparison of the DichroWeb fit–derived secondary structure to the crystal structure 2IHB (Soundararajan et al*.*, 2008) shows relatively good agreement when accounting for possible minute differences between the inactive, GDP-bound state and the active state found in the crystal structure (Figure 4I).

### Fluorescence Polarization Reveals Cholesterol-Induced Modulation of CCR3–CCL11 Affinity

**FIGURE 5:**
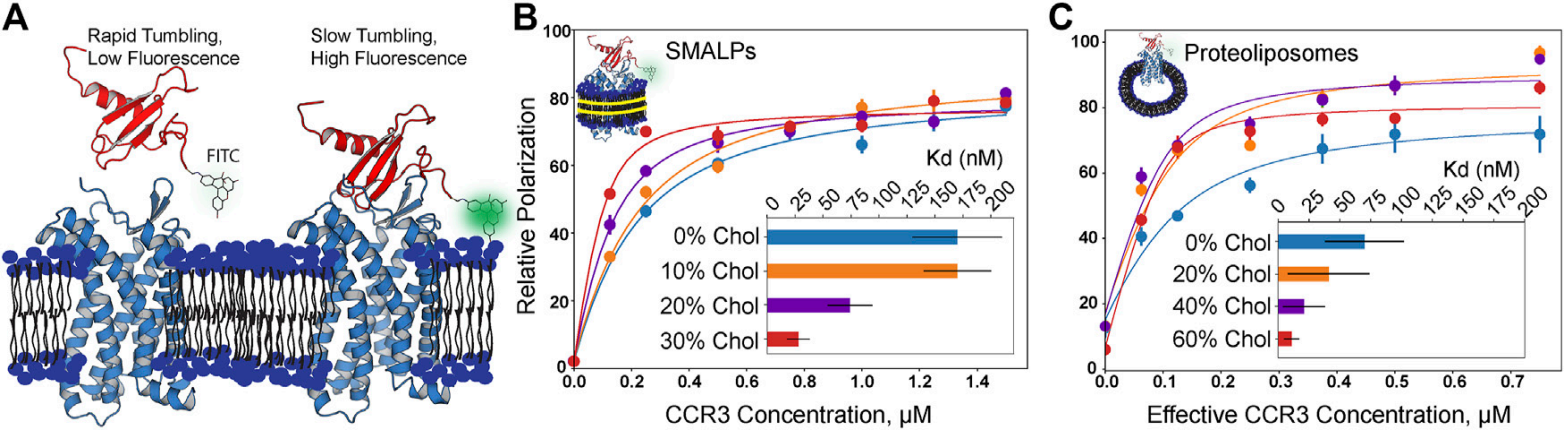
Fluorescence polarization assay results in increasing cholesterol content (mol% in PC). **(A)** Visualization of apo- and CCR3-bound CCL11. FITC-labeled CCL11 is rapidly tumbling in solution, and thus, the probe polarizes light to a lesser degree. Upon binding CCR3, tumbling is slowed, polarizing a greater fraction of light and giving rise to a larger polarization value. CCL11 (PDB ID 1EOT) (Crump et al., 1998) was docked to CCR3 using HADDOCK (van Zundert et al., 2016) to visualize the bound state leading to slower tumbling. **(B)** Membrane cholesterol content is positively correlated with increased ligand affinity in SMALPs. **(C)** Increased observed affinity is conserved in proteoliposomes. Concentration is halved to correct for receptor orientation (Supplementary Figure S3). Points indicate the mean ± SEM for three replicates, each read three times. Error bars may fall within the size of the points. Data were normalized by subtracting the lowest zero-point (no CCR3) value in a curve from each read to bring all curves within the same reference frame.

GPCR structures often contain cholesterol binding motifs, but it is unclear how bilayer cholesterol impacts chemokine receptor function. Thus, we employed fluorescence polarization to measure the affinity of CCR3 for CCL11 as a function of bilayer cholesterol concentration. We attached the fluorophore fluorescein isothiocyanate (FITC) to the *N* terminus of CCL11, which unbound is rapidly tumbling in solution, polarizing light to a lower extent (Figure 5A). After CCL11 is bound, the decreased molecular tumbling increases polarization. Titration of cholesterol from 0 to 30% (mol%) into 1-pamlitoyl-2-oleoyl-phosphatidylcholine (POPC and PC) membrane styrene:maleic acid lipid particles (SMALPs) shows a drastic decrease in Kd (Figure 5B). A Kd of 30 ± 10 nM at 30% cholesterol, or a ∼5-fold decrease from pure POPC, is quite significant.

In order to gauge the effects of cholesterol at higher concentrations, we turned to proteoliposomes, as the membrane rigidity imparted by higher cholesterol content inhibits SMALP formation (Scheidelaar et al., 2015; Dörr et al., 2016) (Figure 5C). It is clear that the same trend is observed; however, the experimental error increased for the measurements in proteoliposomes. We corrected the effective CCR3 concentration to account for receptor orientation intractability by halving the total concentration to remove the statistical average of the receptor with the orthosteric site that is facing into the proteoliposome and thus inaccessible to the ligand. The listed receptor concentrations in proteoliposomes reflect the input concentrations that were halved during curve fitting (Figure 5C; Supplementary Figure S3). Measured Kd values were 20 ± 20 nM in 40% cholesterol and 11 ± 6 nM in 60% cholesterol, following the same inverse trend between Kd and cholesterol content as that seen in SMALPs. However, the Kd values at the same cholesterol concentrations were also lower than the corresponding measurement in SMALPs. Membrane curvature and lateral pressure in the proteoliposomal samples may play a role in GPCR function (Jones et al., 2020). We hypothesize both curvature and lateral pressure are lost in SMALPs, which could contribute to the observed Kd discrepancies between the two conditions. Increasing cholesterol presence did still lead to decreasing Kd in SMALPs, suggesting that curvature and lateral pressure are not the only phenomena responsible for the observed modulation of ligand affinity. Thus, we conclude that cholesterol is a direct positive allosteric effector of CCR3–ligand affinity.

### The Extent of G*α*_i_3 Activation Is Cholesterol Dose Dependent

**FIGURE 6:**
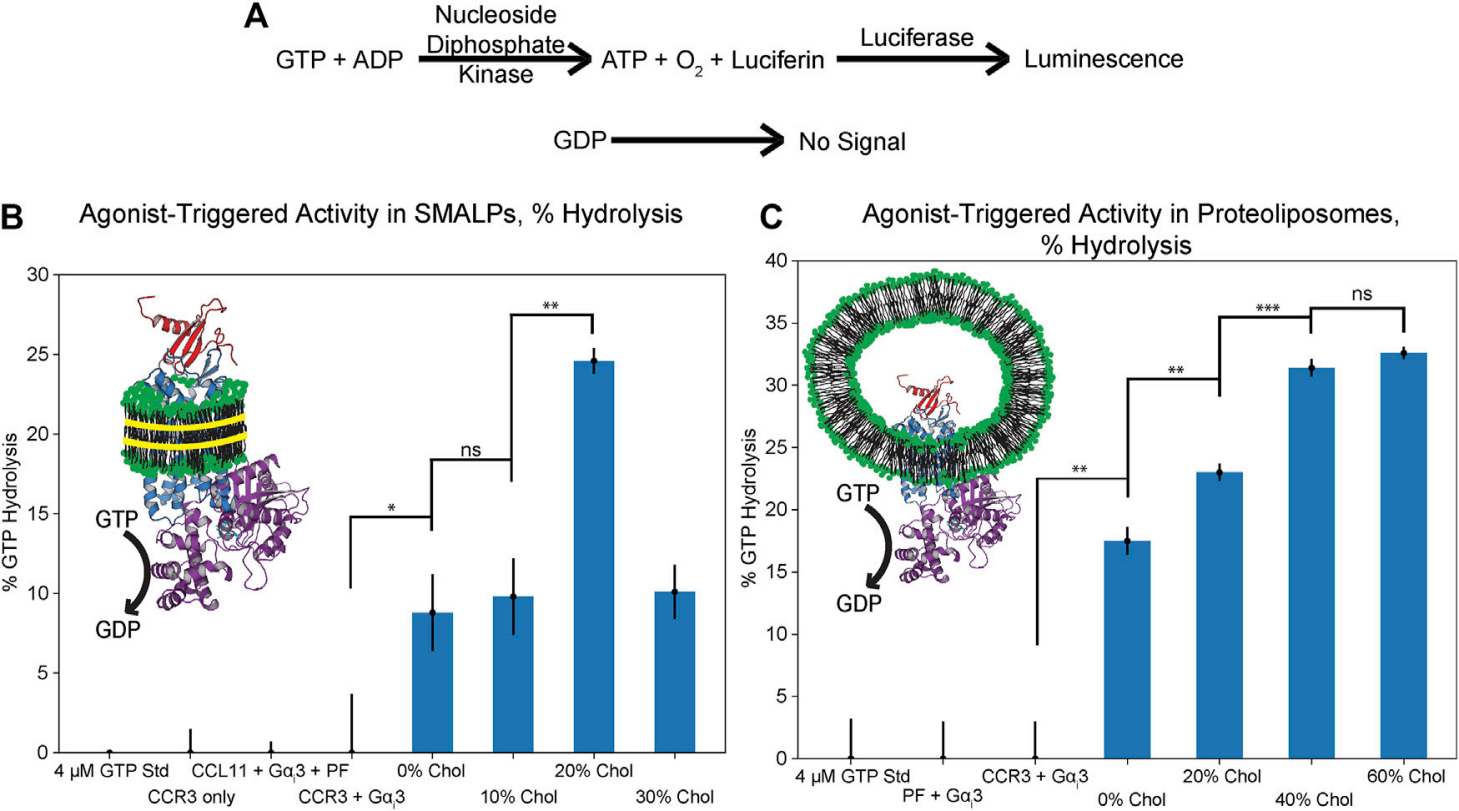
GTPase assay results in SMALPs and proteoliposomes of increasing cholesterol content (mol% in PC), presented as % GTP hydrolyzed over the course of 2 h. **(A)** Schematic representation of GTP hydrolysis translation to the luminescent signal (Mondal et al., 2015). **(B)** Cholesterol enhances GTP hydrolysis in SMALPs but dose dependence could not be verified, likely due to inhibition of disc formation. **(C)** Cholesterol dose dependence of GTP hydrolysis is verified through coreconstitution of CCL11 with CCR3 into proteoliposomes. Cartoons depict CCL11 (red, 1EOT) (Crump et al., 1998) bound to CCR3 (blue) reconstituted in lipids (green headgroups) formed into either SMALPs with SMA (left, yellow) or proteoliposomes (right) with Gα_i_3 (purple, 2ODE) (Soundararajan et al., 2008) bound to CCR3. Points indicate the mean ± standard deviation for three replicates. Bars indicate statistical significance based on the Student’s *t*-test: *p < 0.05, **p < 0.01, ***p < 0.001, ns is not statistically significant.

We next investigated how the observed relationship between cholesterol and CCL11 affinity impacts G protein coupling to CCR3. Heightened activation of CCR3 should lead to increased receptor–G protein coupling. This, in turn, should drive nucleotide exchange in the G*α*_i_3 binding pocket with CCR3 functioning as the guanine nucleotide exchange factor (GEF). The remaining GTP is then converted to a luminescent signal after conversion to ATP (Figure 6A) (Ford et al., 1994; Ford and Leach, 1998a; Ford and Leach, 1998b). In the absence of a suitable GEF, 20 mM EDTA can be used to stimulate intrinsic activity, verifying construct activity (Supplementary Figure S4). We next verified that our CCR3 and G*α*_i_3 constructs are able to couple in detergent, using Mg^2+^ to stabilize the nucleotide-bound state of G*α*_i_3 (Supplementary Figure S5). We considered this an important step as little information is available concerning which Gα_i_ subunits CCR3 activates. Upon showing the ability for our constructs to couple, we investigated the effects of cholesterol on this interaction, hypothesizing that the dose-dependent modulation of agonist affinity would directly translate to G*α*_i_3 coupling, activation, and GTP hydrolysis.

We first attempted to analyze CCL11-driven GTPase activity of G*α*_i_3 in a SMALP environment as both the extracellular agonist orthosteric pocket and the intracellular G protein docking site would be solvent exposed (Figure 6B; Supplementary Figure S6A). While it is clear that cholesterol increases the extent of hydrolysis, dose dependence could not be verified, and the formation and integrity of the cholesterol-containing SMALPs is in question. This is likely due to the rigidity imparted by increasing the cholesterol concentration, which is documented to inhibit copoloymer intercalation within the membrane (Scheidelaar et al., 2015; Dörr et al., 2016). Unlike in the ligand-binding assay, CCR3 immobilized in SMA-resistant cholesterol proteoliposomes is unable to function in this assay regardless of orientation. This may then explain the puzzling decrease in GTPase activity between 20 and 30% datasets. Furthermore, SMA has a relatively narrow range of compatibility with common biochemical assay components such as divalent cations. Thus, CCL11-driven GTPase activity of G*α*_i_3 in SMALPs could not be verified in the presence of Mg^2+^, which also adversely influences G*α*_i_3 GTPase activity (Mondal et al., 2015).

To overcome this challenge, we reconstituted CCR3 in the presence of the peptide agonist. CCR3 was reconstituted into proteoliposomes with 0, 20, 40, and 60% cholesterol content with CCL11 present in the buffer at a molar ratio of 1:5 CCR3:CCL11. The GTPase assay was repeated in the presence of 5 mM Mg^2+^ (Figure 6C; Supplementary Figure S6B). Statistical significance between the GTP hydrolysis signal in 0, 20, and 40% cholesterol is clear evidence of cholesterol dose-dependent modulation of function. Furthermore, GTPase activity in 40 vs. 60% cholesterol proteoliposomes is approximately equivalent, within error, which we also observed in the ligand-binding assay. Together, this corroborates our hypothesis that the dose-dependent modulation of agonist affinity directly translates to receptor activation, G*α*_i_3 coupling, GTP hydrolysis, and thus signal transduction.

## Discussion

Typically, the total exogenous yield of functional GPCRs confounds functional studies. Thus, GPCR production continues to be an area of innovation (Abiko et al., 2021; Mulry et al., 2021). Here, we implemented two techniques, codon harmonization and an MBP fusion tag, to facilitate the production of a functional human GPCR from *E. coli*. Codon harmonization is underutilized ostensibly due to varying degrees of experimental success. This is likely a result of the sheer number of variables that contribute to protein expression and folding (Quax et al., 2015). The use of an MBP fusion tag has also gained popularity, and successful implementation has been observed in a few cases (Bertin et al., 1992; Yeliseev et al., 2007; Serrano-Vega et al., 2008; Egloff et al., 2014; Beckner et al., 2020). Lack of widespread adoption of MBP tags for heterologous GPCR expression is, in our opinion, due to the difficulty of tag removal, as the TEV is known to be inhibited by detergents (Mohanty et al., 2003). This study represents an important step in the successful implementation of a dual heterologous expression strategy that we hypothesize will be of use both in our own future experiments and the wider GPCR structural biology community.

It was shown that the titration of membrane cholesterol increases the affinity of CCR3 for its endogenous ligand CCL11 and that this observation directly translates to agonist-driven G*α*_i_3 GTP hydrolysis as a proxy for signal transduction. We hypothesize that the functional interplay between cholesterol, ligand affinity, and G protein docking is due to cholesterol-driven conformational sampling inhibition. Such observations were made previously, where cholesterol was hypothesized to constrain GPCR conformational selection to states with higher ligand affinity (Weis and Kobilka, 2018). Cholesterol’s influence on chemokine receptor ligand affinity has, however, been shown sparingly (Babcock et al., 2003; Calmet et al., 2020) and typically not dose-dependently. Such observations are likely conserved in chemokine receptors; therefore, we conclude that cholesterol enhances CCR3 ligand binding through a direct allosteric mechanism that is directly translated to G*α*_i_3 coupling and GTP hydrolysis. Thus, our data indicate that cholesterol is a positive allosteric modulator of CCR3 signal transduction.

Although similar fluorescence experiments have been reported (Kawamura et al., 2014; Purvanov et al., 2018; Matti et al., 2020), the use of a recombinant, fluorescently labeled endogenous ligand is nontrivial and may function as a useful tool in future experiments involving *in vitro* mimicry of native biological interactions. Cholesterol as a modulator of agonist-driven GTP hydrolysis of a chemokine receptor is novel to this work. Implementation of SMALP technology is also nontrivial, although the buffer component and lipid incompatibilities still leave room for improvement. Zwitterionic SMALP polymers have broader compatibility with common biochemical reagents and may serve as a starting place in future works (Fiori et al., 2020). This may be the reason this GTPase assay is not more widely adopted in lipid environments, although some experimental evidence is adopted in high-density lipoprotein particles (nanodiscs) (Staus et al., 2019; Huang et al., 2021), and mixed micelles (Gregorio et al., 2017) are evident.

We have shown that G*α*_i_3–CCR3 coupling is possible *in vitro*, but what role this might play *in vivo* is open to speculation. The main cellular function of G*α*_i_ subunits is as intracellular Ca^2+^ ion concentration and adenylate cyclase effectors (Peleg et al., 2002). However, an additional function as modulators of G protein-gated inwardly rectifying K^+^ channels (GIRKs) is well documented (Peleg et al., 2002; Rubinstein et al., 2007). G*α*_i_3, specifically, was originally named G_k_ due to its role in the stimulation of GIRK function (Codina et al., 1987; Codina et al., 1988). A mounting body of evidence now suggests some interplay between chemokine expression and nervous system physiology, with CXCL12, the predominant endogenous ligand of CXCR4, strongly implicated in this phenomenon (Guyon, 2014). Further evidence implicates CXCL12 and CCL5 in the activation of GIRK functionality *in vivo* (Picciocchi et al., 2014), the latter of which is a ligand for CCR3 (Zhang et al., 2015). Thus, there is precedence for chemokine receptor–driven modulation of GIRK functionality. Although predominantly expressed by eosinophils, studies have identified expression of CCR3 in neurons, astrocytes, and microglia (Cho and Miller, 2002; Banisadr et al., 2005). It has been implicated in the pathogenesis of neuroinflammatory conditions such as Parkinson’s disease (Moghadam-Ahmadi et al., 2020) and Alzheimer’s disease (Sui et al., 2019), as well as multiple sclerosis and HIV-associated dementia (Banisadr et al., 2005). Of note, GIRK channels play a direct role in Parkinson’s pathophysiology (Mayfield et al., 2015), and upregulation of serum CCL5 is correlated with disease severity (Tang et al., 2014). Typically, upregulation of CCL5 is related to CCR5 function, but there may be a role for CCL5 and other chemokines triggering CCR3–G*α*_i_3 coupling in neurodegenerative disorders that is certainly worth exploring.

## Data Availability

The original contributions presented in the study are included in the article/Supplementary Material; further inquiries can be directed to the corresponding author.
