# Supplementary material for: Cholesterol Is a Dose-Dependent Positive Allosteric Modulator of CCR3 Ligand Affinity and G Protein Coupling
Source: Front Mol Biosci. 2021 Aug 20;8:724603. doi: 10.3389/fmolb.2021.724603 (PMC8417553; doi:10.3389/fmolb.2021.724603)
Supplement: Supplementary file 1 [file DataSheet1.pdf]

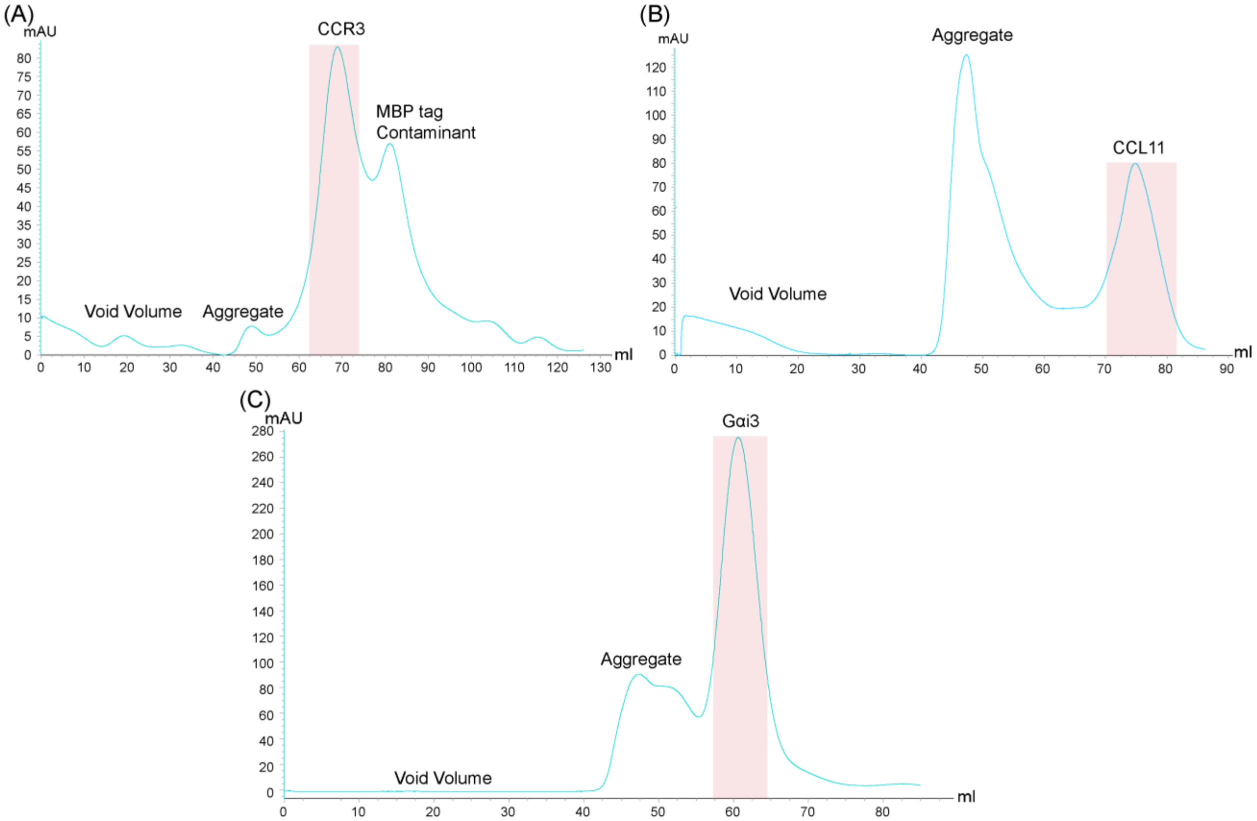

Figure S1. Typical Size Exclusion chromatograms of (A) CCR3, (B) CCL11, and (C) Gα_i3. Red-shaded boxes indicate the typical volume collected, generally performed conservatively to avoid collection of overlapping contaminant peaks.

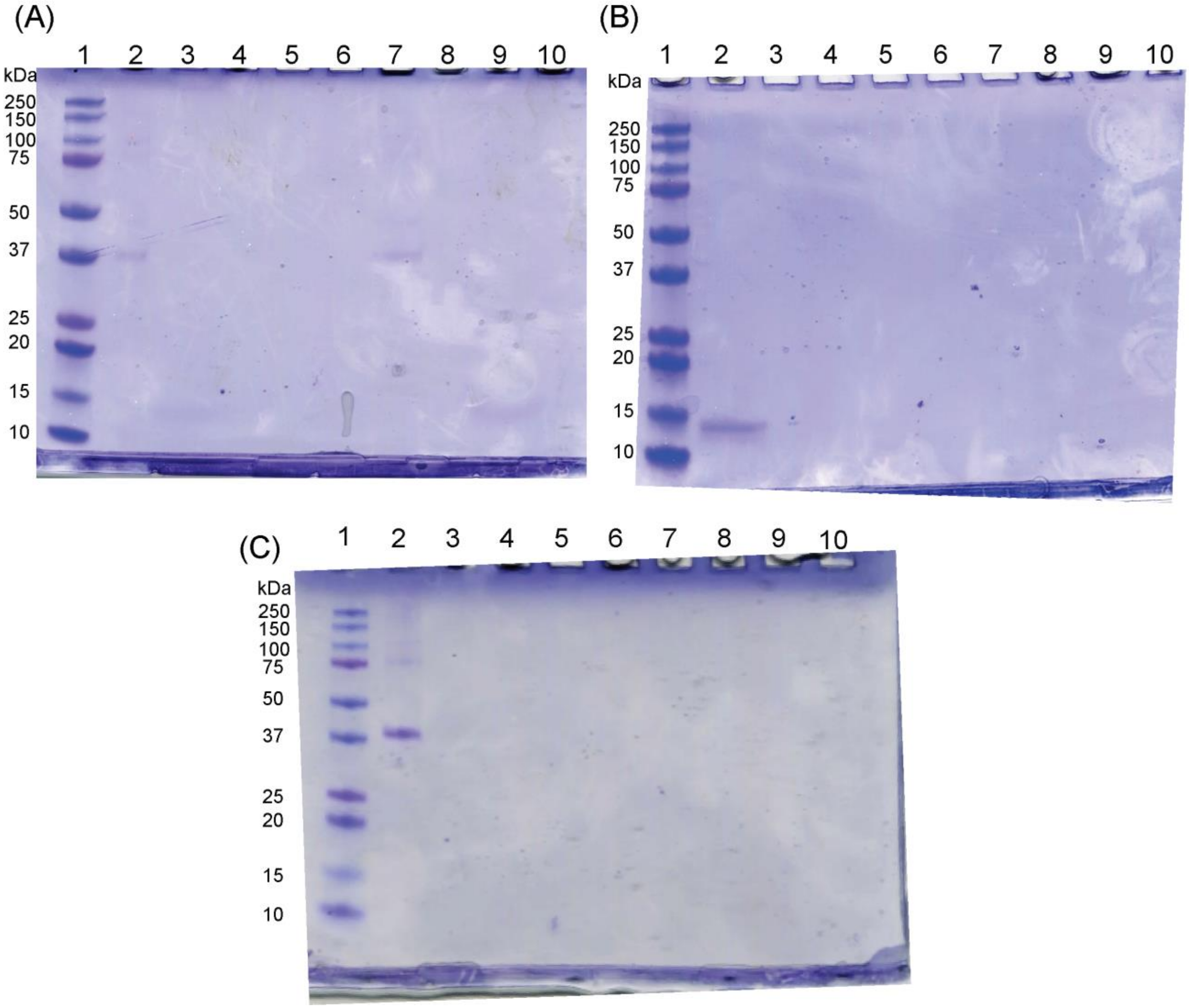

Figure S2. SDS-PAGE full gel images. (A) CCR3 and CCL11 run at different loading volumes with Lane 1: ladder, Lane 2: CCR3, Lane 3: CCL11, Lane 7: CCR3, Lane 9: CCL11. CCR3 loading volumes were constrained by SEC yield, thus Lanes 1 and 2 were chosen for main text presentation. CCL11 was reloaded at a higher concentration in (B) for better visualization. (C) $G\alpha_i3$ band is visible slightly above the 37 kDa marker. Slight contamination is visible at ~75 kDa and ~ 100 kDa.

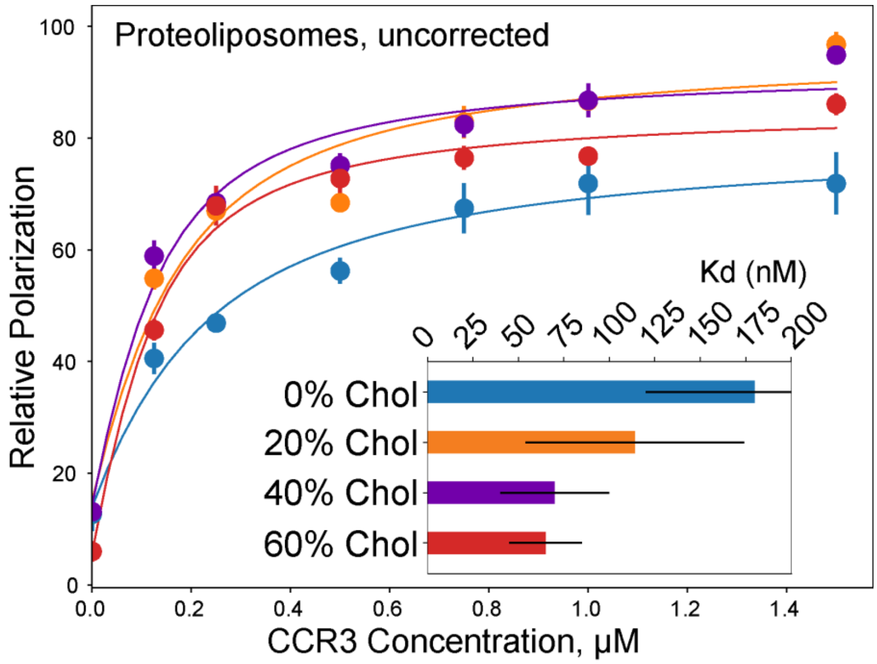

Figure S3. Fluorescence Polarization Analysis in Proteoliposomes. Raw data of CCR3-CCL11 fluorescence polarization binding assay in proteoliposomes with increasing cholesterol content (mol% in PC), uncorrected for receptor orientation. Points indicated the mean ± S.E.M. for three replicates.

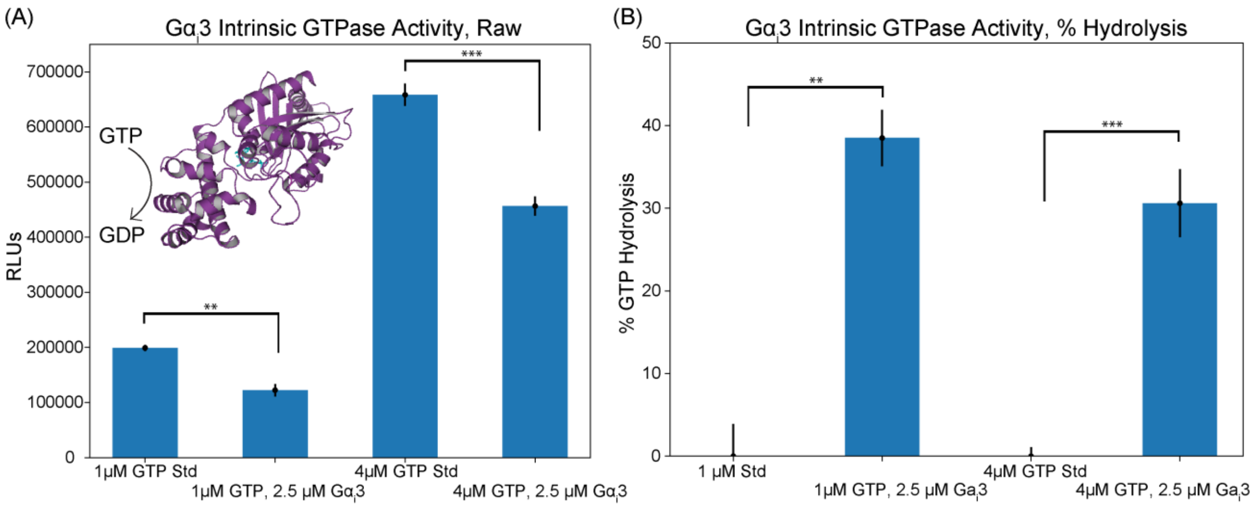

Figure S4. Intrinsic GTPase Activity of Gα$_i$3 measured in (A) raw Relative Light Units (RLUs) using 20 mM EDTA to simulate GEF activity and (B) converted to % GTP hydrolysis. Data was corrected by subtracting the average of 3 blank measurements (no GTP, background luminescence of wells and buffer) from each replicate and is presented as the mean ± standard deviation of three replicates. * denotes $P < 0.05$, ** $P < 0.01$, *** $P < 0.001$, ns is not statistically significant.

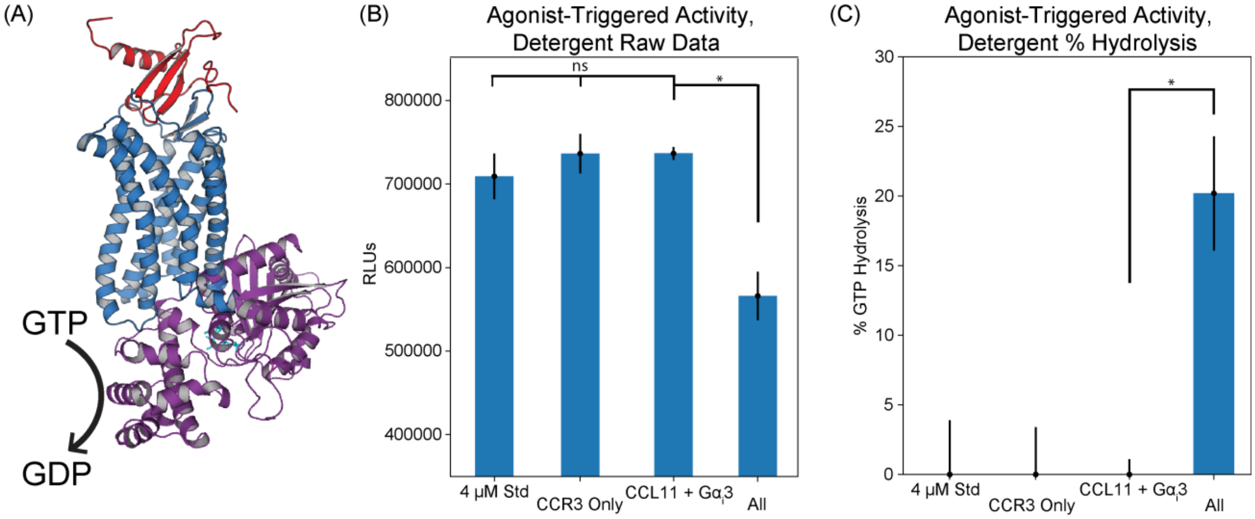

Figure S5. Agonist induced GTPase activity of Gα_i3 in detergent to confirm CCR3-Gα_i3 interaction. (A) Schematic representation of the CCL11-CCR3-Gα_i3 interaction that drives GTP hydrolysis. (B) CCR3-induced GTPase activity in detergent with 1 mM EDTA is apparent only when all 3 required proteins are present, raw Relative Light Units (RLUs). (C) Raw data converted to % GTP hydrolysis. * denotes $P < 0.05$, ** $P < 0.01$, *** $P < 0.001$, ns is not statistically significant.

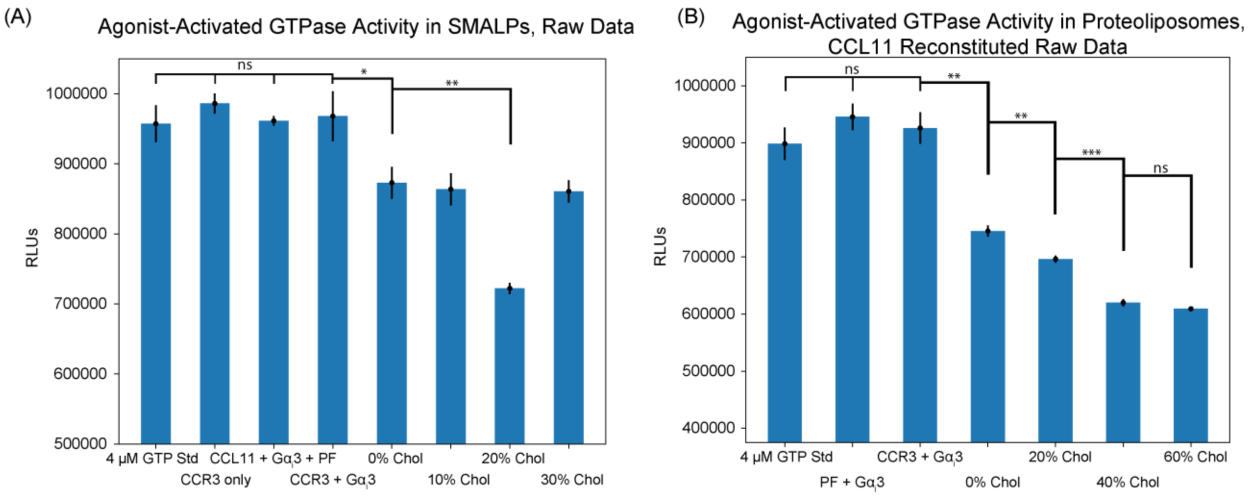

Figure S6. Agonist induced raw GTPase activity of Gαi3 in (A) SMALPs and (B) proteoliposomes of increasing cholesterol content (mol% in PC), Signal is presented as raw Relative Light Units (RLUs).

Table S1: M9 Minimal Media components.

**Solution C, 1 L**	*pH 6.7 with KOH*			
7.3 g KOH	50 ml Metal 44	10 g Nitrilotriacetic acid	24 g $MgCl_2 \cdot 6H_2O$	3.335 g $CaCl_2 \cdot 2H_2O$
**Metal 44 Solution, 100 ml**	*Stored in a dark glass bottle at 4 °C*			
0.327 g $K_2EDTA \cdot 2H_2O$	0.522 g $ZnCl_2$	0.502 g $FeCl_2 \cdot 4H_2O$	0.18 g $MnCl_2 \cdot 4H_2O$	0.0185 g $(NH_4)_6Mo_7O_{24} \cdot 6H_2O$
0.0156 g $CuCl_2 \cdot 2H_2O$	0.0248 g $Co(NO_3)_2 \cdot 6H_2O$	0.0114 g Boric Acid		
